# Aspects of Spirituality in Medical Doctors and Their Relation to Specific Views of Illness and Dealing with Their Patients' Individual Situation

**DOI:** 10.1155/2013/734392

**Published:** 2013-07-17

**Authors:** Arndt Büssing, Almut Tabea Hirdes, Klaus Baumann, Niels Christian Hvidt, Peter Heusser

**Affiliations:** ^1^Institute of Integrative Medicine, Witten/Herdecke University, Gerhard-Kienle-Weg 4, 58313 Herdecke, Germany; ^2^Freiburg Institute for Advanced Studies (FRIAS), Freiburg University, 79104 Freiburg, Germany; ^3^Caritas Science and Christian Social Work, Faculty of Theology, Freiburg University, 79098 Freiburg, Germany; ^4^Institute of Public Health, Faculty of Health Sciences, University of Southern Denmark, 5000 Odense, Denmark; ^5^Chair of Theory of Medicine, Integrative and Anthroposophic Medicine, Institute of Integrative Medicine, Faculty of Health, Witten/Herdecke University, 58313 Herdecke, Germany

## Abstract

We intended to analyse which aspects of spirituality are of relevance for medical doctors in a mostly secular society and how their spiritual/religious attitudes are related to specific views of illness, their dealing with patients' individual situation, and finally physicians' life satisfaction. Data from an anonymous survey enrolling 237 medical doctors from Germany (mean age 45.7 ± 9.6, 58% male, 42% female) indicated that secular forms of spirituality scored highest, while specific religious orientation had the lowest scores. Physicians with a specific specialization in complementary/alternative medicine (CAM) or anthroposophic medicine differed from their conventional counterparts with respect to specific aspects of spirituality; however, the specific views associated with these specialisations were only weakly to moderately correlated with physicians' view on the meaning of illness and how they assume that they would deal with their patients' individual situation. Of interest, the specific aspects of spirituality were negatively correlated with the view of “illness as a meaningless interruption” of life, indicating that physicians with a spiritual attitude would see illness also as a chance for an “individual development” and associated with a “biographical meaning” rather than just a “useless interruption” of life.

## 1. Introduction

Physicians exercise a profession which contains many intrinsic and extrinsic gratifications: intrinsic gratifications like feeling medically competent by acting accordingly, “flow” experiences in being absorbed in their medical activities, achieving good results of treatments, being able to help people in need, being a point of reference, of authority, and of trustworthiness for the patients; as for extrinsic gratifications, we consider social prestige due to their role, financial gains and profits, and positions of superiority. All of these gratifications may in one way or another, in idiosyncratic constellations, help to compensate for the manifold demands and high levels of stress they deal with on a daily scale, both in practice and administration, and also, though less so, in professional politics. “Enough” intrinsic gratifications seem to be more consistent with, and useful for, their satisfaction with their work (and with themselves) rather than a too strong dependence on compensations by extrinsic gratifications [1, 2].

Much research has been done on what motivates people to become doctors [3], how medical students evaluate what makes “a good doctor” [4], and what inspires physicians in their daily work [5]. Likewise, extensive research has looked at the communication between doctors and patients [6], how patients often seek doctors for multiple reasons, many are different from the somatic problem they first present with [7], and how doctors evaluate and seek to alleviate the burdens of their patients [8]. However, there is only limited research on the spiritual/religious attitudes of physicians, and how this may influence the way they interact with their patients.

Among a sample of 1,144 US physicians, 55% would agree that their religious beliefs may influence their practice of medicine [9]. Nevertheless, most would describe themselves as “spiritual” as distinct from “religious,” which contrast with the general US population that sees both concepts “tightly connected” [9]. Most of these US physicians find it “appropriate” to discuss spiritual/religious issues “if the patient brings them up” (91%), and a large fraction encourage “patients' own R/S beliefs and practices” when these issues come up (73%) [10]. Interestingly, particularly physicians with high self-ascribed spiritual/religious attitudes are more likely to inquire spiritual issues and to encourage patients' spiritual/religious beliefs and practices [10]. Further, physicians' specific religious conviction may influence also medical care decision. Curlin et al. [11] reported that particularly highly religious physicians have clear objections to physician-assisted suicide (in fact, the majority of physicians had objections) and also to terminal sedation (which is objected only by 18%) when compared to those with low intrinsic religiosity, while the withdrawal of life support (overall 5% have objections) was not a matter of objection between physicians with low, intermediate, or high religious attitude. This means that specific religious attitudes, convictions, and worldviews of both patients and medical doctors may have an influence on their communication and resulting medical decision [12].

What might be true for the US must not necessarily be true for the more secular Europe. Research has just started in European countries which addresses physicians' spiritual/religious convictions, and how these may influence their practice of medicine.

We thus intended to analyse (1) which aspects of spirituality are of relevance for medical doctors in a mostly secular society and (2) whether and how these spiritual/religious attitudes are related to specific views of illness, their dealing with patients' individual situation, and finally physicians' life satisfaction. In particular, we intended to know whether physicians with a specific focus on complementary and alternative medicine (CAM) approaches differ from conventional physicians. For this purpose, we decided to choose a more open approach to address a wide variety of important aspects of spirituality, both religious and secular forms.

## 2. Materials and Methods

### 2.1. Participants

In this anonymous survey, we enrolled 237 medical doctors recruited via the university's network of family practitioners, in different hospitals and wards with which medical students were in contact (convenience sample with a response rate of 59%). Because we intended to compare physicians with a specific focus on complementary and alternative medicine (CAM) approaches with conventional physicians, we specifically recruited physicians also in hospitals known to offer CAM treatments on the one hand, and physicians in conventional hospitals and wards.

All were assured of confidentiality, consented to participate, and completed the questionnaire, which neither requested names nor initials, by themselves. We had neither inclusion nor exclusion criteria (just the will to participate).

### 2.2. Measures

#### 2.2.1. Aspects of Spirituality

To measure a wide variety of important aspects of spirituality beyond conventional conceptual boundaries, we developed a questionnaire on the basis of the answers of expert representatives of various spiritual orientations which aspects of spirituality are relevant to them (i.e., Catholics, Protestants, members of the Anthroposophic “Christengemeinschaft”, Bahá'í, Muslims, Jews, Buddhists, and atheists) [13]. We condensed the identified motifs to 40 items of the original ASP questionnaire [14] which primarily differentiates 7 factors (Cronbach's alpha = .94), that is, *prayer, trust in God, and shelter*; *insight, awareness, and wisdom*; *transcendence conviction*; *compassion, generosity, and patience*; *conscious interactions*; *gratitude, reverence, and respect*; and *equanimity*. For this analysis, we used a shortened version with 25 items (ASP 2.1) which differentiates 6 factors [15], that is,

1. *religious orientation* (9 items; alpha = .93), that is, praying, feeling guided and sheltered, trust in and turn to God, spiritual orientation in life, distinct rituals, reading spiritual/religious books, and so forth,
2. *search for insight/wisdom* (7 items, alpha = .88) with two subconstructs:
  - *aspiring beauty/insight *(4 items; alpha = .76), that is, developing wisdom, aspiring to insight and truth, aspiring to beauty and goodness, and aspiring to broad awareness,
  - *quest orientation* (3 items; alpha = .76), that is, life is a search and question for answers, search for deep insight in fabric of life, and achieve of frankness/wideness of the spirit;
3. *conscious interactions* ****(5 items, alpha = .83) with two sub-constructs:
  - *conscious interaction* (3 items, alpha = .75), that is, with others, self, and environment,
  - *compassion/generosity* (2 items, alpha = .63), that is, developing compassion and practicing generosity;
4. *transcendence conviction* (4 items, alpha = .85), that is, belief in the existence of higher beings, rebirth of man/soul, soul origins in “higher” dimensions, and man is a spiritual being.

All items were scored on a 5-point scale from disagreement to agreement (0—does not apply at all; 1—does not truly apply; 2—does not know (neither yes nor no); 3—applies quite a bit; 4—applies very much). The scores are referred to a 100% level (4 “applied very much” = 100%).

#### 2.2.2. Life Satisfaction

Life satisfaction was measured with the *Brief Multidimensional Life Satisfaction Scale* (BMLSS) [16] which refers to the Huebner's “Brief Multidimensional Students' Life Satisfaction Scale” [17, 18]. The eight items of the BMLSS address intrinsic dimensions (*Myself*, *Overall life*), social dimensions (*Friendships*, *Family life*), external dimensions (*School situation*, *Where I live*), and the prospective dimension (*Financial situation*, *Future prospects*). The internal consistency of the instrument was good (Cronbach's alpha = .87) [16]. Each item was introduced by the phrase “I would describe my satisfaction with …” and scored on a 7-point scale from dissatisfaction to satisfaction (0—terrible; 1—unhappy; 2—mostly dissatisfied; 3—mixed (about equally satisfied and dissatisfied); 4—mostly satisfied; 5—pleased; 6—delighted). The BMLSS sum score was referred to a 100% level (“delighted”).

*Single Items Addressing Physicians' Dealing with Their Patients and Meaning of Illness. *To address physicians' self-perceived dealing with their patients' individual situation and their view about how illness may impact patients' life in terms of “meaning,” we used 10 single statements (see [19]), that is, Item SK1 “whether a patient may see any meaning in illness and life or not is not of importance for the process of recovery,” Item SK2 “to me, it is completely incomprehensible that illness may have a biographical meaning in the life of man,” Item SK3 “illness is nothing more than a meaningless interruption of the course of life,” Item SK4 “illness prevents patients' individual development,” Item SK5 reverse “illness is a chance to deal more consciously with life,” Item SK9 “I have no time to busy myself with patients' individual situation,” Item SK10 “if I had also to deal with patients' individual situation, it would unnecessarily cost time and nerves,” Item SK6 “whether a patient may understand the profound causes of illness or not is irrelevant for the process of recovery,” Item SK8 “often it is simply a matter of fate or chance whether a patient becomes healthy again or not,” and Item ÄS18 “for diagnosis and finding of an adequate treatment, patients' own opinion about what may have caused their illness is not of importance.”

All items were scored on a 5-point scale from disagreement to agreement (0—does not apply at all; 1—does not truly apply; 2—does not know (neither yes nor no); 3—applies quite a bit; 4—applies very much).

### 2.3. Statistical Analyses

Descriptive and analyses of variance and first-order correlations and regression analyses were computed with SPSS 20.0. Given the exploratory character of this study, significance level was set at *P* < 0.05 when mean scores were compared and at *P* < 0.01 when correlations between the respective variables were analysed. With respect to classifying the strength of the observed correlations, we regarded *r* > .5 as a strong correlation, an *r* between .3 and .5 as a moderate correlation, an *r* between .2 and .3 as a weak correlation, and *r* < .2 as no or a negligible correlation.

## 3. Results

### 3.1. Demographic Results

Among the 237 medical doctors with a mean age of 45.7 ± 9.6, 58% were male and 42% female, 81% were living with a partner and 19% alone, and a Christian denomination was predominating (Table 1).

With respect to their academic status, 61% had a doctoral graduation, 3% had a postdoctoral lecture qualification, and 36% had a medical diploma. As shown in Table 1, 44% were registered doctors and 54% working in a hospital (32% as assistant physicians and 22% as senior physicians). Among them, 29% were working in the field of general medicine, 25% internal medicine, 13% surgery/orthopaedic, 8% gynaecology, 7% anaesthesia, and 6% paediatrics. Their life satisfaction was high (Table 1).

### 3.2. Aspects of Spirituality in Medical Doctors

The highest spirituality scores were found for *conscious interactions* and *compassion/generosity*, followed by *aspiring beauty/wisdom*, *quest orientation*, and *transcendence convictions*, while *religious orientation* had the lowest scores indicating indecisiveness (Table 2).

Nevertheless, the physicians are active in specific religious activities; that is, 47% are praying for others (46% not, 7% undecided), and 37% are praying for themselves (54% not, 9% undecided); 46% are reading spiritual texts/scriptures (37% not, 7% undecided), and 32% are meditating (58% not, 10% undecided).

Women had significantly higher scores only for *conscious interactions* (Table 2), while all other ASP scores did not significantly differ (Table 2). Moreover, aspects of spirituality did not differ with respect to the age (categories 25–40 years, 41–50 years, 51–69 years; *F* values range from 0.3 to 2.1; n.s.) or family status (*F* values range from 0.2 to 2.2; n.s.). Those with a Christian denomination had significantly higher scores for *religious orientation *(*F* = 33.7; *P* < 0.0001), *transcendence conviction* (*F* = 30.5; *P* < 0.0001), *aspiring beauty/wisdom* (*F* = 7.0: *P* = 0.001), and *quest orientation* (*F* = 4.1; *P* = 0.017), and in trend also for *compassion/generosity* (*F* = 2.4; *P* = 0.091), but not for *conscious interactions* (*F* = 0.0; n.s.).

Also the academic grade had no significant impact on the spirituality scores (*F* values range from 0.0 to 1.3; n.s.). However, within the field of the medical profession, *religious orientation* (*F* = 2.4; *P* = 0.024) and also *transcendence conviction *(*F* = 2.1; *P* = 0.043) showed significant differences, which could be explained in part by the underlying specialisations (i.e., complementary medicine and anthroposophic medicine versus conventional medicine).

#### 3.2.1. Meaning of Illness and Dealing with Patients' Individual Situation

As shown in Table 3, most medical doctors would clearly reject the statement that “it is not of importance for the process of recovery whether a patient may see any meaning in his illness and life or not” (Item SK1), that it is “completely incomprehensible that illness may have a biographical meaning” (SK2), that “illness is nothing more than a useless interruption of the course of life” (SK3), and that “illness prevents patients' individual development” (SK4), while most would agree that “illness is a chance to deal more consciously with life” (SK5). These 5 items form a unique factor with good internal consistence (Cronbach's alpha = .81) termed “illness as a meaningless interruption” (with inversely coded item SK5) which would explain 57% of variance and can be used in further analyses.

Five further statements address physicians' dealing with the individual situation of their patients. Here, the responses were less clear-cut (Table 3). While most would disagree that they have “no time to become busy with patients' individual situation” (SK9: 67% disagreement), 23% agreed and 10% were unclear. However, the strict statement “if I had also to deal with patients' individual situation, it would unnecessarily cost time and nerves” (SK10) was rejected by 88% (7% agreement, 8% undecided). This means that most clearly intend to address patients' individual situation and regard it as essential.

In line with this, most rejected the statement “whether a patient may understand the profound causes of illness or not is irrelevant for the process of recovery” (SK6: 83% rejection), while the statement that “patients' own opinion about what may have caused their illness is not of importance for diagnosis and finding an adequate treatment” (ÄS18) was commented positively by a minority of physicians (10% agreement, 67% rejection, and 13% undecided). This means that most have an implicit perception that patients' individual perspectives should be included in the process of diagnosis and treatment. However, the more fatalistic statement that it is often “a matter of fate or chance whether a patient becomes healthy again or not” (SK8) showed the strongest variance; that is, 45% rejected this statement, while 27% agreed, and 28% were undecided.

Particularly medical doctors with a specialisation in AM and psychotherapy rejected the statement that “illness is nothing more than a useless interruption of the course of life” (SK3) more strictly than conventional physicians. Due to their specific spiritual orientation and worldview, AM doctors clearly rejected the statement that “illness prevents patients' individual development” (SK4) or that it is “completely incomprehensible that illness may have a biographical meaning” (SK2); conventional physicians would reject these statements too but in a less clear-cut manner.

All other statements did not show significant differences with respect to medical doctors' professional specialisation or underlying orientation (Table 3).

### 3.3. Associations between Aspects of Spirituality, Life Satisfaction, and Specific Statements

Next we intended to analyse associations between aspects of spirituality, life satisfaction, and statements towards illness, treatment and recovery (Tables 4 and 5).

Aspects of spirituality were strongly interconnected, particularly *transcendence conviction* and *religious orientation* and the scales *conscious interactions* and *search for insight/wisdom *with their subconstructs. Interestingly, *conscious interactions* and *religious orientation* were only weakly associated. However, life satisfaction was associated weakly and negatively only with *conscious interactions *and *aspiring beauty/wisdom *(Table 4), but with none of the other aspects of spirituality. Partial correlations with the variable CAM specification are presented in Table 5.

All aspects of spirituality were inversely related to the negative statements that “illness prevents patients' individual development” (SK4) and that “illness is nothing more than a useless interruption of course of life” (SK3). The opposite statement that illness “may have a biographical meaning” (SK2) was thus positively associated particularly with *conscious interactions* and *compassion/generosity* and just weakly with the other aspects of spirituality (Table 4). The respective factor “Illness as a Meaningless Interruption” thus correlated moderately and negatively with the specific aspects of spirituality, particularly with transcendence conviction (Table 4).

The specific statements addressing physicians' dealing with their patients' individual situation were in most cases either not at all or only weakly associated with their spirituality. However, particularly the strict statement “if I had also to deal with patients' individual situation, it would unnecessarily cost time and nerves” (SK10) was negatively associated with the relational forms of spirituality (i.e., *conscious interactions*). Of particular interest, there were no significant correlations between physicians' specific aspects of spirituality and the conviction that patients' process of recovery is a matter of fate or chance (SK8) and the time physicians' think they have to invest in their patients' individual situation (SK9).

## 4. Discussion

Among the recruited medical doctors, particularly secular and relational forms of spirituality were of relevance (i.e., *conscious interactions* and *compassion/generosity*), while the more specific religious forms were of the lowest relevance. This is in line with the findings among adolescents/young adults with a high-school education in secular society such as that of Germany [15], also in patients with chronic diseases, engagement in religious practices was the lowest when compared to secular forms of practice [20, 21].

Of specific interest was the fact that particularly physicians with an unconventional specialisation in CAM and AM had significantly higher score on the different aspects of spirituality when compared to physicians with either no or conventional specialisations. Thus, one could suggest that this unique fact may have (1) either an influence on the way they care for their patients and (2) how they would interpret illness and its impact on patients' course of life.

While the majority of physicians regard illness as a patients' chance to deal more consciously with life (92%), and only a minority would have a negative or indecisive perception of illness (up to 18%), it was of interest that the specific aspects of spirituality were negatively correlated with the view of “illness as a meaningless interruption” of life. This means that physicians with a spiritual attitude would see illness also as a chance for an “individual development” and associated with a “biographical meaning” rather than just a “useless interruption” of life which was rejected by 88%. However, particularly those with an AM background rejected the point of view that “illness is nothing more than a meaningless interruption of life's course” and that “illness prevents patients' individual development” very strongly. This strict and unique point of view can be explained by the conviction that illness and other forms of hardship in life can (but do not have to) be “tasks in life,” the experience of which may even contribute to the inner development of an individual [22].

With respect to the physicians' perception how they think they are dealing with patients' individual situation, only the relational and existential forms of secular spirituality were negatively and only weakly associated with the cynical statement that dealing with patients' individual situation “would unnecessarily cost time and nerves” and that patients' individual points of view in regard of the suggested causes of illness “is irrelevant for the process of recovery”. With respect to these patient-oriented views, physicians with a CAM or AM specialisation and those with a conventional specialisation did not differ significantly.

While psychosomatic medicine has already called for a medical ethos of physicians which overcomes the so-called value neutrality of the 19th century (see [23], 597ff.), more recent German textbooks on “good doctors” and “good medical care” either plea for a basic philosophical attitude of the physician committed to respect the otherness of the other [24] or to put the human person into the center of any medical practice [25]. Religious bases or spiritual aspects are only marginally alluded to or remain implicit. There is an evident shyness regarding explicit religious or spiritual elements which may be essential to physicians' professional identity and fulfilment [26] or which, however, might influence medical decisions and behaviour. This is probably due to religion and spirituality dealing with transcendent realities and thus transcending the borders of normal scientific, evidence-based, science on which modern health care is thought to be based [27]. This is—to some extent—different in the field of palliative medicine and care, also due to the explicit mention of spiritual aspects in the 2002 WHO-definition. Data of Geiss and Belschner [28] indicate that transpersonal trust is an important resource for German medical doctors working in palliative/intensive care units, senior residential homes, and hospices. Although most of the physicians analysed herein were working in a conventional medical context and not in palliative care units, adequate communication and interest in patients' concerns should not be restricted to patients with severe handicaps, cancer, or hospices.

The development of “spiritual care,” in consequence, is still mainly restricted to the end of life medicine [29, 30], although first steps are being taken to go beyond and acknowledge the empirically tested relevance of spiritual and religious needs, values, and attitudes of both patients and clinical staff in other fields of medicine like psychiatry and psychotherapies [31] and chronic illnesses [32].

However, a limitation of the study is the fact that the data are not representative for German physicians. This was not our intention because we aimed to oversample medical doctors with a specific specialisation in CAM (including AM) to compare their data with conventional medical doctors. Moreover, we are aware that particularly the items addressing the suggested meaning of illness may be answered with social desirability, and thus the focus of attention should be on the undecided and those who agreed to the respective statement (8–18%). The same is true for two items addressing their perceptions and how they deal with patients' individual situation (i.e., items SK6 and SK10 with positive and undecided answers in 12–17%).

## 5. Conclusion

While the physicians with a specific CAM or AM specialisation differ from their conventional counterparts with respect to specific aspects of spirituality, the specific views associated with these specialisations were only weakly to moderately associated with their view on the meaning of illness and how they assume that they would deal with their patients' individual situation. On the other hand, physicians' spirituality was either not at all or only weakly associated with their life satisfaction, particularly their conscious interactions with others, self, and environment (which is not a specific religious issue), and their aspiration to develop wisdom, insight and truth, beauty and goodness, and broad awareness is of relevance (which is at least moderately associated with a religious orientation). Nevertheless, it was of interest that the specific aspects of spirituality were negatively correlated with the view of “illness as a meaningless interruption” of life, indicating that physicians with a spiritual attitude would see illness also as a chance for an “individual development” and associated with a “biographical meaning” rather than just a “useless interruption” of life. 

## Figures and Tables

**Table 1:** Demographic and psychometric data of 237 medical doctors.

Mean age (mean: years)	45.7 ± 9.6
Gender (%)	
Male	58
Female	42
Family status (%)	
Married	68
Living with partner	13
Divorced	8
Single	11
Denomination (%)	
Christians	79
Other	5
None	16
Academic grade (%)	
Professor	3
Doctor	61
Diploma	36
Physician status (%)	
Assistant physician (hospital)	32
Senior physician (hospital)	22
Registered doctor	44
Specialisation (%)	
CAM (naturopathy, TCM, and homeopathy)	20
Anthroposophic medicine (AM)	19
Psychotherapy	12
None/conventional	50
Life satisfaction (mean: 0–100)	77.4 ± 12.8

**Table 2:** Aspects of spirituality (main scales and respective subscales) in physicians.

	Religious orientation	Quest orientation	Aspiring beauty/wisdom	Conscious interactions	Compassion/generosity	Transcendence conviction
	Action/emotion	Action (intention)	Action/intention	Action	Intention	Cognition
*All physicians *						
Mean	49.2	74.7	75.0	83.4	81.6	66.6
SD	25.5	20.6	18.0	14.2	15.0	27.5

*Gender *						
Female						
Mean	51.07	75.86	76.72	86.25	83.25	68.34
SD	27.22	22.42	18.40	13.18	14.64	26.00
Male						
Mean	48.16	74.04	74.00	81.60	80.56	65.37
SD	27.89	19.67	17.80	14.58	15.01	28.50
*F* value	0.6	0.4	1.3	**6.2**	1.9	0.7
*P* value	n.s.	n.s.	n.s.	**0.013**	n.s.	n.s.

*Specialisation *						
CAM						
Mean	43.61	72.87	72.07	89.72	82.98	60.90
SD	31.58	22.75	18.79	11.55	16.37	29.67
AM						
Mean	67.91	87.10	86.41	88.29	88.10	92.56
SD	17.68	12.24	12.08	11.50	12.33	15.01
Psychotherapy						
Mean	45.50	81.09	78.53	83.33	84.62	61.30
SD	28.63	15.91	16.65	16.83	17.43	25.74
None/conventional						
Mean	45.94	69.65	71.37	79.38	78.23	60.97
SD	25.95	21.50	18.10	14.22	14.10	25.38
*F*-value	**8.6**	**9.0**	**8.7**	**8.6**	**5.3**	**18.4**
*P*-value	**<0.0001**	**<0.0001**	**<0.0001**	**<0.0001**	**0.001**	**<0.0001**

Scores > 50% indicate a positive attitude, while scores < 50 indicate a rejection or disagreement.

**Table 3:** Meaning of illness and physicians' dealing with patients' individual situation with respect to professional specialisation.

		Agreement/disagreement (%)			Professional specialisation (means ± SD)				
		Yes (applies quite a bit; applies very much)	Undecided	No (does not apply at all; does not truly apply)	CAM	AM	Psychotherapists	None/ conventional	*F* value (*P* value)
	*Illness as a meaningless interruption* (*Sum scores, range 0–100*)	—	—	—	14.7 ± 14.5	7.5 ± 9.6	10.0 ± 11.7	19.5 ± 17.4	**7.8 ** **(<0.001)**

	*Meaning of Illness* (range 0–4)								
SK1	Whether a patient my see any meaning in illness and life or not is not of importance for the process of recovery	4	6	90	0.77 ± 0.81	0.43 ± 0.63	0.52 ± 0.77	0.75 ± 0.86	2.1 (0.095)
SK2	To me it is completely incomprehensible that illness may have a biographical meaning in life of man	4	4	92	0.55 ± 0.88	0.17 ± 0.66	0.32 ± 0.63	0.57 ± 0.87	3.0 (0.033)
SK3	Illness is nothing more than a meaningless interruption of life's course	4	8	88	0.43 ± 0.69	0.05 ± 0.31	0.16 ± 0.37	0.74 ± 0.95	**10.3 (<0.001)**
SK4	Illness prevents patients' individual development	7	11	82	0.52 ± 0.62	0.14 ± 0.35	0.40 ± 0.58	1.04 ± 1.09	**13.7 (<0.001)**
SK5	Illness is a chance to deal more consciously with life	92	4	4	3.34 ± 0.76	3.29 ± 0.84	3.40 ± 0.71	3.20 ± 0.78	0.7 (n.s.)

	*Dealing with patients' individual situation* (range 0–4)								
SK9	I have no time to busy myself with patients' individual situation	23	10	67	1.19 ± 1.25	1.43 ± 1.15	1.12 ± 1.20	1.45 ± 1.15	0.9 (n.s.)
SK10	If I had also to deal with patients' individual situation, it would unnecessarily cost time and nerves	7	5	88	0.57 ± 1.04	0.69 ± 0.90	0.44 ± 0.71	0.85 ± 0.91	2.0 (n.s.)
SK6	Whether a patient may understand the profound causes of illness or not is irrelevant for the process of recovery	**7**	10	83	0.78 ± 0.70	0.85 ± 0.88	0.52 ± 0.77	0.94 ± 0.93	1.7 (n.s.)
SK8	Often it is simply a matter of fate or chance whether a patient becomes healthy again or not	27	28	45	1.47 ± 1.28	1.90 ± 1.11	1.68 ± 1.14	1.68 ± 1.11	1.0 (n.s.)
ÄS18	For diagnosis and finding an adequate treatment, patients' own opinion about what may have caused their illness is not of importance	10	13	67	1.09 ± 1.15	0.88 ± 0.86	0.59 ± 0.97	1.03 ± 0.98	1.7 (n.s.)

**Table 4:** Intercorrelations of aspects of spirituality in medical doctors.

	Religious orientation	Quest orientation	Aspiring beauty/wisdom	Conscious interactions	Compassion/generosity	Transcendence conviction
*Aspects of spirituality *						
Religious orientation		.473**	.486**	.169**	.291**	.743**
Quest orientation			.693**	.449**	.424**	.557**
Aspiring beauty/insight				.365**	.402**	.515**
Conscious interactions					.531**	.291**
Compassion/generosity						.347**

*Life satisfaction (Sum Score) *	.139	.083	.230**	.271**	.126	.040

*Illness as a meaningless interruption (sum score) *	−.304**	−.332**	−.259**	−.399**	−.381**	−.406**

*Meaning of illness *						
Item SK1 “whether a patient my see any meaning in illness and life or not is not of importance for the process of recovery”	−.185**	−.179**	−.123	−.273**	−.280**	−.189**
Item SK2 “to me it is completely incomprehensible that illness may have a biographical meaning in life of man”	−.237**	−.262**	−.240**	−.347**	−.361**	−.292**
Item SK3 “illness is nothing more than a meaningless interruption of life's course”	−.258**	−.376**	−.301**	−.387**	−.392**	−.409**
Item SK4 “illness prevents patients' individual development”	−.314**	−.395**	−.334**	−.386**	−.375**	−.461**
Item SK5 reverse “illness is a chance to deal more consciously with life”	−.223**	−.254**	−.178**	−.279**	−.240**	−.290**

*Dealing with patients' individual situation *						
Item SK9 “I have no time to busy myself with patients' individual situation”	−.035	−.087	−.112	−.156	−.060	−.027
Item SK10 “If I had also to deal with patients' individual situation, it would unnecessarily cost time and nerves”	−.084	−.200**	−.184**	−.332**	−.225**	−.153
Item SK6 “whether a patient may understand the profound causes of illness or not, is irrelevant for the process of recovery”	−.088	−.251**	−.128	−.213**	−.256**	−.123
Item SK8 “often it is simply a matter of fate or chance whether a patient becomes healthy again or not.”	.154	−.031	.046	−.123	−.159	.046
Item ÄS18 “for diagnosis and finding an adequate treatment, patients' own opinion about what may have caused their illness is not of importance”	−.204**	−.210**	−.134	−.237**	−.183**	−.160

**P < 0.01 (Spearman rho).

**Table 5:** Intercorrelations of aspects of spirituality in medical doctors (partial correlation analysis).

Controlled for “CAM specialization”	Religious orientation	Quest orientation	Aspiring beauty/wisdom	Conscious interactions	Compassion/generosity	Transcendence conviction
Aspects of spirituality						
Religious orientation		.497	.497	.200	.317	.779
Quest orientation			.720	.462	.400	.586
Aspiring beauty/insight				.344	.372	.529
Conscious interactions					.545	.303
Compassion/generosity						.341
Life satisfaction (sum score)	.164	.113	.213	.268	.125	.075
Illness as a meaningless interruption (sum score)	−.291	−.401	−.300	−.429	−.408	−.405

**P < 0.01 (partial correlation; controlled for CAM specialization yes/no).
